# Cardiovascular risk factors among Chamorros

**DOI:** 10.1186/1471-2458-6-298

**Published:** 2006-12-08

**Authors:** Binh Chiem, Victoria Nguyen, Phillis L Wu, Celine M Ko, Lee Ann Cruz, Georgia Robins Sadler

**Affiliations:** 1 Moores UCSD Cancer Center, La Jolla, California, USA; 2 Department of Surgery, University of California, San Diego School of Medicine, La Jolla, California, USA; 3 University of California, San Diego School of Medicine, La Jolla, California, USA; 4 Pacific Islander Cancer Control Network, Office of Chamorro Affairs, Inc., San Diego, California, USA

## Abstract

**Background:**

Little is known regarding the cardiovascular disease risk factors among Chamorros residing in the United States.

**Methods:**

The Chamorro Directory International and the CDC's Behavioral Risk Factor Surveillance System Questionnaire (BRFSS) were used to assess the health related practices and needs of a random sample of 228 Chamorros.

**Results:**

Inactivity, hypertension, elevated cholesterol and diabetes mellitus were more prevalent in this Chamorro sample compared to the US average. Participants who were 50-and-older or unemployed were more likely to report hypertension, diabetes and inactivity, but they were also more likely to consume more fruits and vegetables than their younger and employed counterparts. Women were more likely to report hypertension and diabetes, whereas men were more likely to have elevated BMI and to have never had their blood cholesterol checked.

**Conclusion:**

The study provides data that will help healthcare providers, public health workers and community leaders identify where to focus their health improvement efforts for Chamorros and create culturally competent programs to promote health in this community.

## Background

Cardiovascular disease, which encompasses heart disease, stroke and hypertension, was responsible for 16.7 million deaths in 2003. This number represented one-third of total deaths worldwide [1]. In the United States, cardiovascular disease alone is responsible for approximately 1 in every 5 American deaths [2]. It is currently the number one cause of death in developed countries, and the World Health Organization (WHO) projects that by 2010, cardiovascular disease will also be the major cause of morbidity and mortality in developing countries [1].

Cardiovascular disease is the leading cause of death across all ethnic groups, including Caucasians, African-Americans, Hispanics, American Indians/Alaskan Natives. Among Asian Americans and Pacific Islanders (AAPI) [2,3] it is virtually tied with cancer as the leading causes of death. Compared to other ethnic groups, the frequency of cardiovascular disease is relatively low in the AAPI community [4]. This has resulted in a relative lack of research of the AAPI populations as a focus group among public health efforts to prevent or delay cardiovascular disease [5]. The groups within the AAPI community, such as the Chamorro community which is discussed in this paper, have received even less attention.

Cardiovascular disease is a major cause of morbidity and mortality within the AAPI community, accounting for over 25% of all AAPI deaths in 2001 [3]. Complicating the assessment of cardiovascular disease and health promotion interventions in the AAPI community is the way that Census data were collected for the Asian and Pacific Islander community. Prior to the 1990 Census, Asians and Pacific Islanders were aggregated into a single group. While the 1990 Census allowed Asians and Pacific Islanders to select a subgroup for membership, it did not allow respondents to select multiple sub-groups, a particular problem for a community where a high proportion of people are multiracial. For the 2000 Census, it was possible for respondents to select multiple racial categories and thereby more accurately depict their racial heritage [6]. Certain subgroups within the AAPI conglomerate suffer more from cardiovascular disease than others. For example, data collected by the Missouri State Health Department showed that overall, AAPI mortality from heart disease is half the rate of Caucasians, and one-third the rate of African-American. However, closer scrutiny of the AAPI data, reveals that the Hawaiian subgroup, for example, has mortality rates 2.4 times that of Caucasians and 1.6 times that of African-American [7]. The AAPI population is highly heterogeneous. Understanding the specific variations that exist in the backgrounds, cultural practices, and morbidity and mortality rates of AAPI subgroups will facilitate the creation of optimal health interventions that can be "narrow casted" to those AAPI sub-groups known to have the greatest risk of developing premature cardiovascular disease [8]. Narrowcasting of health promotion messages involves the development of a social marketing campaign that is to be focused on a narrowly defined audience [9-11].

Thus to develop a niche social marketing campaign, it is first necessary to understand the audience, the outcomes to be accomplished, and the messages to be delivered toward this end. In this study, Chamorros and cardiovascular disease were the focus of the research team. While Pacific Islanders comprise only 0.3% of the total U.S. population [12], they are the second fastest growing ethnic group after Asians at-large. The Census Bureau projections indicate that by 2050, the number of Pacific Islanders living in the United States will have tripled [13]. Thus it is imperative that adequate public health information be gathered about this group so that optimal health promotion intervention can be provided to this growing community.

The Pacific Islands are divided into three large geographical regions: Micronesia, Polynesia, and Melanesia. Chamorros are indigenous people of the Mariana Islands, a sub-division of Micronesia. The vast majority of this ethnic group is found in Guam, an island of the Marianas archipelago, and the continental United States. According to the 2000 Census, of those Chamorros living in the continental United States, more than one-third resided in California, with San Diego County home to the largest Chamorro community outside of Guam [14,15]. Thus, San Diego is an ideal site for a study of the continental U.S. Chamorro population. The health needs of the Chamorros have often been overshadowed by the larger, more visible AAPI ethnicities. An exhaustive search of the scientific literature yielded little information regarding the health status of the Chamorro people residing on the continental United States, especially in relation to cardiovascular disease [16-22]. Even in the 2001 Center for Disease Control and Prevention, National Center for Health Statistics (CDC/NCHS) publication, no reliable data were reported for the incidence of cardiovascular disease, even in the larger category of Pacific Islanders [4].

In this paper, the authors report on the risk factors for cardiovascular disease that were identified in San Diego's Chamorro community. By documenting such baseline data in this rapidly growing minority group, health care providers and community leaders can identify and create culturally competent, specific interventions for Chamorros.

## Methods

### Subject recruitment

The Center for Disease Control and Prevention's 2001 core Behavioral Risk Factor Surveillance System (BRFSS) [23] and five of 14 optional modules were used to survey participants. The national data gathered using the 2001 BRFSS were used as the reference point to which the data collected from the Chamorro community was compared.

The *Chamorro Directory International *(a telephone directory composed exclusively of people who self-identified as members of the Chamorro community) was used to reach members of the local Chamorro community. Leaders of the Chamorro Community have maintained the Directory since 1995, updating and reprinting it every 5 years. Data is gathered by word-of-mouth requests for entry information and more recently, by Internet solicitations. As identifiable information is received, every potential entry is contacted to confirm the accuracy of the information and to secure written approval to publish the information. Using a University of California San Diego IRB-approved protocol, all 658 names in the *Chamorro Directory International *[24] for the local region were entered into a computerized database. Then a computer-generated call list was created to determine who would be called and in what order they would be called. Calls were made until at least 100 men and 100 women from the Chamorro community had been recruited to the study. The sample size was limited by the modest size of the planning grant ($10,000) that was received to help the community conduct a health assessment. The first adult to answer the phone was invited to complete the survey. The invitation to participate included the IRB-approved verbal informed consent script which included a strong statement of confidentiality. If he/she refused to participate, other household members were invited and likewise consented. Of those households contacted, the individual response rate was 62.8%. To obtain a sample diverse in age and socioeconomic status, calls were made seven days a week, from early morning until late evening. The survey workers were bilingual members of the Chamorro community. The community newspaper and a word-of-mouth campaign by the Pacific Islander Cancer Control Network (PICCN) leaders helped encourage participation in the study.

A midpoint review of the sample's socio-demographic data indicated an under-representation of men and male and female Chamorros less than 50 years of age. To address this problem, the recruitment strategy was modified such that surveyors randomly asked to speak with the oldest adult, youngest adult, or any adult male in the family throughout the remainder of the data collection process. Completed surveys were individually reviewed for specific health problems and risk factors. Based on their perceived needs, participants were mailed a packet of personalized health promotion information such as preventive care and self-help resources. At the end of the project, a second packet was mailed containing a letter describing the project's findings and information regarding the community's most pressing overall health risks and needs. All transactions were mailed first-class, so that undelivered mail could be tracked. Only one packet of the 250 mailed was returned as undeliverable. Three of the 228 participants requested that no packet be mailed. All were given this opportunity.

The collected data were entered into an ACCESS 2000 database and analyzed with SPSS for Windows 10.0 [25]. Chi-squared tests were used to establish relationships between socioeconomic characteristics and cardiovascular disease risk factors. A p-value of < 0.05 was considered statistically significant. Risk factors were analyzed by gender, age (< 50 versus ≥ 50), and employment status to determine whether differences existed within those sample subsets.

Frequencies of the eight known risk factors for cardiovascular disease (BMI, physical activity, diet, hypertension, blood cholesterol, diabetes, smoking, and alcohol consumption) were also contrasted with the American Heart Association data [26,27].

## Results

### Sample description

This study sample consisted of 100 male and 128 female Chamorros from San Diego County. The male participants ranged in age from 18 to 87 years. Average age for the men was 48.7 years (SD = 17.7); the median, 47.0. The female participants ranged in age from 19 to 88 years. Average age for the women was 54.7 years (SD = 15.2); the median, 57.5. The median education category for this sample group was "1 to 3 years of college," with a range from grade school to at least four years of college. More than half of the study participants withheld information regarding household income, so all results related to income should be interpreted with caution. Of the 49.6% who reported an income, the median household income was "$35,000 – $50,000." Women were much more willing than men to volunteer their income data: 63% of female versus only 33% of male participants reported their income. The vast majority (93%) of participants had healthcare coverage, while a large portion (47%) reported excellent/very good health. Most of the participants were middle-aged (47%), married (61%), and had no children living in the household (62%). Participants' sociodemographic data are presented in Table 1.

### Eight risk factors of cardiovascular disease

#### 1. Body Mass Index (BMI)

According to the National Heart, Lung and Blood Institute (NHLBI), an ideal BMI ranges from 18.5 to 24.9. Any person with a BMI of 25 to 29.9 is considered overweight, while a BMI of 30 and greater is considered obese [28]. In this study sample, the BMI was not available for 21% of the participants as 28 males and 20 females declined to provide either their height or weight. Of those who reported their height and weight, the average BMI was 27.4 (male 28.0, female 27.0) with a range of 17.2 to 47.7 (male 18.3 – 44.1, female 17.2 – 47.7) and an overall standard deviation of 5.6 (male 4.4, female 6.3). The majority of study participants were either overweight or obese: 31.1% were overweight while an additional 21.1% were obese (Table 2). Men were significantly more likely than women to be overweight or obese (p < .05). Participants who were employed were much more likely than the unemployed to be overweight or obese: (p < .05). Higher income also correlated with an elevated BMI (0r = .250, p < .05).

#### 2. Physical activity

Physical activity was calculated as number of days/week multiplied by amount of time/day a person spent doing either moderate or strenuous physical exercise. The CDC recommends a minimum of 150 minutes of moderate exercise or 60 minutes of vigorous exercise per week [29] and the American Heart Association's (AHA) recommended 90 minutes of moderate physical activity per week. Among the participants, 66.7% did less than 150 minutes of moderate exercise per week, 67.4% did less than 60 minutes of vigorous exercise per week, and 34% did not exercise at all (Table 2). Participants who were 50 and older or those not employed were at significantly higher risk of failing the AHA guideline (p < .05).

#### 3. Diet

Daily consumption of fruits and vegetables was calculated for each participant. Total fruit included fruit and fruit juices, whereas total vegetables included green salad, non-fried potatoes, carrots, and others. The AHA's Food Pyramid recommends eating at least 3 servings of vegetable and 2 servings of fruit per day [30]. Sixty percent of the study participants failed to consume the minimum of 5 servings of vegetable and fruit per day (Table 2). Participants who were younger than 50 or employed outside of the home tended to eat less fruit or vegetable than their older and unemployed counterparts (p < .05).

#### 4. Hypertension

Of the 228 study participants, 42.5% reported they had been diagnosed with hypertension, but only 34.2% reported taking medication for their hypertension (Table 2). Females, those aged 50 and over, and those unemployed were diagnosed with hypertension at significantly greater frequencies than their male, younger, and employed counterparts (p < .05). Among the female participants hypertension was associated with less education, but this trend was not seen in the male participants. Less educated women reported hypertension at a much greater frequency than their more educated counterparts (p < .05).

#### 5. Blood cholesterol

The AHA recommends that all adults starting at age 20 should obtain a full lipid profile once every five years [31]. Seventy percent of study participants reported compliance, but 27% never had blood drawn for a lipid panel (Table 2). This is especially true among the male participants. Significantly more men than women had never had their blood cholesterol checked (p < .05). Those participants who were male, aged less than 50, and employed were significantly more likely than their counterparts to have never checked their blood cholesterol (p < .05).

#### 6. Diabetes

Of those Chamorros surveyed, 16.2% were diabetic, and an additional 2.6% reported gestational diabetes (Table 2). The mean age of diagnosis for men was 58.0 years (median age 60, SD = 11.3) and 46.1 years for women (median 50, SD = 9.1). Participants who were female, aged 50 and above, and not employed were significantly more likely to have diabetes than those who were male, younger than 50, and employed (p < .05).

#### 7. Tobacco use

Fifteen percent of study participants were smokers. Half (51%) of those who smoked had tried to quit within the last year (Table 2). The average age for first time smoking cigarettes was 14.9 years (± 4.2 SD) for men and 19.1 years (± 7.8 SD) for women. The average age when they first started smoking regularly was 16.8 years (± 4.2 SD) for men and 20.9 years (± 5.9 SD) for women. Forty-one participants (21 men and 20 women) reported smoking at least 100 cigarettes in their lifetime but were current nonsmokers. The survey also queried about smoking restrictions encountered at home and in the workplace. Within the home, 83% of the 35 smokers indicated they could *not *smoke inside the house at all, 3% could smoke in certain designated areas or at certain times, 6% could smoke anywhere, and 8% had no rules about smoking inside the home. Of those same 35 smokers, 57% worked indoors, and 80% of these indoor workers were prohibited from smoking in public and work areas.

#### 8. Alcohol consumption

The daily consumption of alcoholic beverages was calculated for each participant as the number of days per month in which one had at least one drink, multiplied by the average number of drinks consumed on each of those days, then divided by 30 to obtain a daily average. The AHA recommends drinking should be limited to an average of one to two drinks per day for men, and one drink per day for women [32]. Out of the 228 participants, only four averaged more than one drink per day, and most (63.6%) had not drunk at all in the month preceding the survey (Table 2).

### Cumulative risk factors

The mean number of cumulative cardiovascular disease risk factors (CRF) for this study population was 2.01 per person. As seen in Table 3, 35% had at least three CRF. Participants who were 50 years and older, unemployed, and less educated reported substantially higher CRF than their younger, employed, and more educated counterparts (p < .05).

### Weight management

Table 4 shows that more than half of the study participants were actively managing their weight: 25% were trying to lose weight, 25% were trying to maintain current weight, and 5% responded "yes" to both options. Women were more likely than men to report that they were trying to lose or maintain their weight (p < .05). Of those 128 individuals trying to manage their weight, 16 (13%) used exercise only, 25% (32) used diet only, and 57% (73) used both diet and exercise to achieve their goals. In total, 105 dieted and 89 exercised (overlap exists where participants reported both dieting and exercising). Of those 105 who dieted to control their weight, 18% (19) cut back on calories only, 37% (39) cut back on fat only, and 45% (47) chose to reduce both calories and fat intake. Participants aged 50-and-above preferred to lose weight by dieting, whereas younger participants under-50 preferred exercising (p < .05). Those who had a BMI of 25 and above reported they were trying to lose weight with much greater frequency than those with a lower BMI (p < .05). When queried about an ideal weight, 122 of the 228 participants responded. Of those respondents, 6 wanted to gain weight, 26 wanted to maintain their current weight, and 86 wanted to lose weight. Among those who wanted to lose weight, the mean amount of weight loss desired was 28.60 lbs (24.44 lbs for men, 30.31 lbs for women) with a range of 2 lbs to 150 lbs. The "desired weight" reported by each participant was converted to a "desired BMI" based on the individual's height. As shown in Table 4, the ideal weight reported by most respondents fell within the healthy BMI range of 20 to 25. However, when examined by gender, most (57) female respondents desired a BMI of 20 to 25, whereas most (24) male respondents desired a higher BMI of 25 to 30.

## Discussion

Cardiovascular disease is the number one cause of death in the U.S., making it a major national health threat with far-reaching consequences. Since individual health behaviors are highly predictive of disease development and mortality, an effective disease prevention campaign begins with an assessment of modifiable disease behaviors. This study provides an assessment of the cardiovascular disease-related health behaviors of San Diego's Chamorro population. The data can be used by health care providers, public health advocates, and indigenous community leaders to develop cardiovascular disease prevention and intervention strategies that are culturally specific, sensitive, and appropriate to this AAPI ethnic subgroup.

While the data in this study has not been age-adjusted, compared to state and national data [33] the Chamorros in this study appeared more susceptible to four of the eight risk factors: low physical activity, hypertension, high blood cholesterol, and diabetes. Chamorros (66.7%) appear to be less likely to adhere to the CDC's weekly recommendation for moderate exercise compared to state (53.3%) and national (52.8%) averages. Hypertension and diabetes were present among the Chamorros at an alarming rate. Chamorros in this study reported hypertension (42.5%) at a much greater frequency than other Californians (23.4%) and nation-wide numbers (24.8%). The prevalence of diabetes among Chamorros (16.2%) was more than twice the state (7.2%) and national (7.1%) figures. Gestational diabetes among Chamorros (2.6%) reflected similarly disproportionate figures compared to the state (1.1%) and national (0.7%) rates. In addition, Chamorro women reported high blood cholesterol (40.6%) more frequently than the average Californian woman (30.5%) and national average (32.1%). In contrast, the prevalence of high blood cholesterol was slightly lower in Chamorro men (31.0%) than their state (35.1%) and national (33.8%) counterparts.

As expected, the sociodemographic characteristics of age, gender, and employment status were often aligned with the behavioral risk factors. Participants who were 50 years and older and unemployed tended to report hypertension, diabetes, and low physical activity, but they also consumed more fruits and vegetables than their younger and employed counterparts. Women were more likely than men to be hypertensive and diabetic; but men were more likely than women to have an elevated BMI and to have never had their blood cholesterol checked. Men were more likely than women to have set a optimal weight goal that would result in an unhealthy BMI. These are clearly opportunities for interventions by primary care health providers, public health policy makers, the media, and community leaders. Women started smoking later than men, at an age (19.1 years ± 7.8 SD) which coincides with the beginning of gynecology care. This may indicate a role for gynecologists in the primary prevention of smoking in Chamorro women. Conversely, the fact that male participants started smoking at a much younger age (14.9 years ± 4.2 SD) indicates the need for earlier intervention in the male Chamorro population.

Among the eight behavioral risk factors examined, BMI is considered to be of great importance due to its relationship to the other risk factors. There has been discussion within the scientific community as to whether the BMI used in the U.S. is applicable to other ethnic minority groups. Some research suggests that there are bone size differences among various ethnic groups that make the simple calculation using height and weight recordings less accurate than a calculation that includes bone size considerations. Further, while these studies suggest that ethnic specific cut-off points for BMI would be more appropriate, there are no widely accepted standards for Pacific Islanders. Therefore, for this community health assessment, the standard BMI cut-off point was necessarily used.

Reducing BMI has also been shown to reduce one's risk of developing diabetes, hypertension, and elevated blood cholesterol. Given the immense health benefits of BMI reduction to patients, it is important to ensure patients' related health knowledge and access to supportive services. If health professionals can broach and discuss the topic in a culturally sensitive manner, patients will be more receptive to recommendations and also more likely to cooperate. When helping patients plan a weight loss program, it is important to set attainable goals. The concept of an ideal weight is inherently related to ideal body image, which in turn is determined partly by culture. It is a well-known fact that body image varies widely among cultures. Fat and obesity carry a powerful stigma in modern-day Western society, but are held in high regard among many of the Pacific Islander traditional cultures [34]. Among Hawaiians and Samoans, for example, corpulence was dignifying and indicative of wealth [35,36]. A cross-cultural comparison between Australian and Samoan women indicated overweight Samoan women felt healthier and more attractive than their Australian equivalents [37]. One study comparing body size perceptions between different ethnic groups living in New Zealand revealed that Pacific Islanders most frequently underestimated their body weight [38]. These findings may indicate that among Pacific Islanders, being overweight or obese is more socially acceptable. Since continental US Chamorros are descendants of the Pacific Island heritage, they may also possess this cultural propensity for obesity. To be effective counselors, health professionals should first determine how the patient perceives his or her own body weight currently, and then ascertain that an "ideal weight" in the clinical sense matches the patient's expectations.

Once a patient's weight and fitness goals have been established, the next step is to devise a weight loss and exercise program that will fit into the community's cultural structure. An earlier paper that assessed this community's risk for diabetes began to address the cultural challenges health care providers will need to be sensitive to, as they strive to help the community improve its over all health status [18]. Diet has an important cultural context among Chamorros. Researchers have cited Western influence on the native Chamorro's diet as a cause of adverse health effects. A study of children's diet in Guam documented a drastic change from the traditional vegetarian and fish diet to a Westernized, meat-based diet consisting of fatty and processed food [21]. Chamorro populations with increasing Western influence were found to eat a higher-fat diet and suffer higher coronary heart disease mortality [19]. Chamorros living in the continental US also experience this "Westernization." Health educators can take advantage of cultural pride by encouraging Chamorro patients to modify their diet toward the more traditional ethnic diet of plants and fish [18]. It has been postulated that Westernization has made a greater impact on Pacific Islanders' diet and lifestyle than it has on their cultural perspectives on body image [34]. The basis for this statement arises from a study of Samoans' body size perception where, although the Samoan defined thinner bodies as ideal, which reflected a definite Western influence, they were not overtly concerned with striving for this ideal [39]. Overweight individuals were quite satisfied with their large body size, and obese Samoan women were no more likely to try and lose weight than their thinner counterparts. The same may also hold true for continental US Chamorros, and the resulting health effects can be detrimental.

The Chamorro leaders who helped to initiate this community health assessment have demonstrated their ability to undertake a campaign to improve the health of their community. Through focus groups and community town hall meetings, as well as the exchange of information with other Chamorro communities, these same leaders can next begin to evaluate the universality of these findings. Where appropriate, they can also begin to develop strategies to address identified problems or develop plans for further investigation. The interventions they develop are likely to be closely aligned with the values and social fabric of their community. To be optimally resourceful in their program development, Chamorro leaders will need to call upon available public health educators and clinicians to help them interpret the latest medical literature to assure that the health promotion goals they set are grounded on evidence-based research.

### Limitations

This study is only the first step in the process of understanding how to optimize the health and well being of the Chamorro community. This study has definite limitations as regards the generalizability of the findings, including the potential for self-selection bias (62.8% response rate), the use of self-reported information that could not be verified in person, the small sample size, the unwillingness of those contacted to report about household income and body mass index and the narrow geographic eligibility for the sample. However, as an exploratory study, the information gathered and analyzed can lay the groundwork for the development of future studies and testable interventions to address those behaviors which increase this community's risk of cardiovascular disease.

#### Lessons for the international scientific community

This baseline health risk assessment for the Chamorro community was initiated by the leaders of the local Chamorro community to identify and address the factors that contribute to improving and diminishing the health of their community. It is a demonstration of a community-campus partnership in action. Through this collaboration with members of the local scientific community, the Chamorro leaders now have preliminary knowledge to help their members gain more personalized insight into their cardiovascular risk status and how to improve it. In turn, the University and the Chamorro community leaders now have a solid partnership that they can use as a foundation for future mutually beneficial undertakings.

## Conclusion

Healthcare providers play a crucial role in the crusade against cardiovascular disease. Their frequent interactions with patients give them the opportunity to alter the course of this deadly illness by stressing behavioral risk reductions and other prevention strategies. Increasing awareness of the health needs of the Chamorro community and providing culturally competent intervention will enhance health providers' effectiveness as counselors with this ethnic group. Chamorro leaders have an equally important role to play in guiding their community toward the adoption of optimally healthful life styles. This paper presents baseline cardiovascular disease-related health data specific to the continental U.S. Chamorro population. It also relates various Pacific Islander cultural values and practices that may affect Chamorro patients' responsiveness to health professionals.

## Competing interests

The author(s) declare that they have no competing interests.

## Authors' contributions

All of the authors read and approved the final manuscript.

## Pre-publication history

The pre-publication history for this paper can be accessed here:



## Figures and Tables

**Table 1:** Participants' Sociodemographic Data

**Participants' Characteristics**		**BRFSS results for Chamorro Men n = 100 (%)**	**BRFSS Results for Chamorro Women n = 128 (%)**	**Total n = 228 (%)**
**Age Range**	18–39	31 (31.0%)	22 (17.2%)	53 (23.2%)
	40–65	39 (39.0%)	69 (53.9%)	108 (47.4%)
	66+	21 (21.0%)	23 (18.0%)	44 (19.3%)
	Refused	9 (9.0%)	14 (10.9%)	23 (10.1%)
**Marriage Status**	Married	56 (56.0%)	84 (65.6%)	140 (61.4%)
	Not Married*	37 (37.0%)	43 (33.6%)	80 (35.1%)
	Refused	7 (7.0%)	1 (0.8%)	8 (3.5%)
**Number of children in household**	None	62 (62.0%)	80 (62.5%)	142 (62.3%)
	Any children	37 (37.0%)	47 (36.7%)	84 (36.8%)
	Refused	1 (1.0%)	1 (0.8%)	2 (0.9%)
**Education Completed**	Less than HS	1 (1.0%)	5 (3.9%)	6 (2.6 %)
	HS or GED	28 (28.0%)	53 (41.4%)	81 (35.5 %)
	College 1–3 yrs	58 (58.0%)	54 (42.2%)	112 (49.1%)
	College 4 yrs +	12 (12.0%)	16 (12.5%)	28 (12.3%)
**Employment Status**	Employed/Self Employed	63 (63.0%)	57 (44.5%)	120 (52.6%)
	Unemployed	37 (37%)	71 (55.5%)	108 (47.4%)
**Household Income**	Less than 15 K	1 (1.0%)	5 (3.9%)	6 (2.6%)
	15 K – 25 K	4 (4.0%)	15 (11.7%)	19 (8.3%)
	25 K – 35 K	5 (5.0%)	8 (6.3%)	13 (5.7%)
	35 K – 50 K	11 (11.0%)	16 (12.5%)	27 (11.8%)
	50 K +	12 (12.0%)	36 (28.1%)	48 (21.1%)
	Don't know/not sure/refused	67 (67.0%)	48 (37.5%)	115 (50.4%)
Health Coverage***	Yes	91 (91.0%)	122 (95.3%)	213 (93.4%)
	No	9 (9.0%)	6 (4.7%)	15 (6.6%)
**Self-Rated Health Status**	Excellent/very good	56 (56%)	51 (39.8%)	107 (46.9%)
	Good	26 (26%)	45 (35.2%)	71 (31.1%)
	Fair/Poor	18 (18%)	31 (24.2%)	49 (21.5%)

*Not married – divorced, widowed, separated, never married, member of an unmarried couple

**Unemployed – out of work, homemaker, student, retired, unable to work

***Health coverage – health insurance, prepaid plans, or government plans such as Medicare

**Table 2:** Cardiovascular Disease Risk Factors

	**Chamorro Men n = 100(%)**	**Chamorro Women n = 128 (%)**	**Total n = 228 (%)**
Body Mass Index (BMI)*			
BMI 25.0 – 29.9 (Overweight)	36 (36.0%)	35 (27.3%)	71 (31.1%)
BMI ≥ 30 (Obese)	20 (20.0%)	28 (21.9%)	48 (21.1%)
**Physical Activity**			
Moderate exercise for less than 150 min/wk	71 (71.0%)	81 (63.3%)	152 (66.7%)
Vigorous exercise for less than 60 min/wk	59 (59.0%)	95 (74.2%)	154 (67.4%)
Moderate exercise for 0 min/wk	44 (44.0%)	39 (30.5%)	83 (36.4%)
Vigorous exercise for 0 min/wk	56 (56.0%)	93 (72.7%)	149 (65.3%)
Do not exercise at all (moderate or vigorous)	39 (39.0)	38 (29.7%)	77 (33.8%)
**Diet**			
Eat less than 2 servings of fruit/day	53 (53.0%)	55 (43.0%)	108 (47.4%)
Eat less than 3 servings of vegetable/day	57 (57.0%)	76 (59.4%)	133 (58.3%)
Eat less than 5 servings of fruit or veg./day	62 (62.0%)	74 (57.8%)	136 (59.6%)
**Hypertension (HTN)**			
Told by a health professional they have HTN	35 (35.0%)	62 (48.4%)	97 (42.5%)
Currently taking medication for HTN	30 (30.0%)	48 (37.5%)	78 (34.2%)
**Blood Cholesterol**			
Never checked	39 (39.0%)	23 (18.0%)	62 (27.2%)
Checked within past 5 years	61 (61.0%)	98 (76.6%)	159 (69.7%)
Told by a health professional they have high blood cholesterol	31 (31.0%)	52 (40.6%)	83 (36.4%)
**Diabetes**			
Diagnosed with diabetes	12 (12.0%)	25 (19.5%)	37 (16.2%)
Gestational diabetes	--	6 (4.7%)	6 (2.6 %)
**Tobacco Use**			
Current smoker **	19 (19.0%)	16 (12.5%)	35 (15.4%)
Have tried to quit in the past 12 months	10 (10.0%)	8 (6.3%)	18 (7.9%)
Current non-smoker***	59 (59.0%)	91 (71.1%)	150 (65.8%)
**Alcohol Consumption**			
Average >1 drink/day	3 (3.0%)	1 (0.8%)	4 (1.8%)
Average >2 drinks/day	2 (2.0%)	1 (0.8%)	3 (1.3%)
No drinks in past 30 days	58 (58.0%)	87 (68.0%)	145 (63.6%)

*Body Mass Index (BMI) = weight (lbs)/height (in)^2^* 703

**Smoker – one who has smoked at least 100 cigarettes (5 packs) in his/her lifetime and who is smoking some days or everyday.

***Non-smoker – one who has *not *smoked at least 100 cigarettes (5 packs) in his/her lifetime and is *not *currently smoking some days or everyday.

**Table 3:** Chamorro Cumulative Risk Factors

**Cumulative Risk Factors (CRF)**	**Chamorro Men n = 100 (%)**	**Chamorro Women n = 128 (%)**	**Total n = 228 (%)**
**0 RF**	14 (14.0%)	3 (10.2%)	27 (11.8%)
**1 RF**	27 (27.0%)	39 (30.5%)	66 (28.9%)
**2 RF**	28 (28.0%)	28 (21.9%)	56 (24.6%)
**3 RF**	16 (16.0%)	29 (22.7%)	45 (19.7%)
**≥ 4 RF**	15 (15.0%)	19 (14.8%)	34 (14.9%)

**Table 4:** Weight Control

	**Chamorro Men n = 100(%)**	**Chamorro Women n = 128 (%)**	**Total n = 228 (%)**
**WEIGHT STATUS**			
Advised to lose weight by a health professional within the past 12 months	6 (6.0%)	22 (17.2%)	28 (12.3%)
Trying to maintain current weight only	19 (19.0%)	39 (30.5%)	58 (25.4%)
Trying to lose weight only	20 (20.0%)	38 (29.7%)	58 (25.4%)
**PREFERRED WEIGHT CONTROL TECHNIQUE**			
Lose/Maintain current weight by dieting:			
**YES**	30 (30.0%)	75 (58.6%)	105 (46.1%)
Eat fewer calories only	4 (4.0%)	15 (11.7%)	19 (8.3%)
Eat less fat only	8 (8.0%)	31 (24.2%)	39 (17.1%)
Eat fewer calories and less fat	18 (18.0%)	29 (22.7%)	47 (20.6%)
**NO**	13 (13.0%)	15 (11.7%)	28 (12.3%)
Lose/Maintain current weight by exercising:			
**YES**	32 (32.0%)	57 (44.5%)	89 (39.0%)
**NO**	11 (11.0%)	33 (25.8%)	44(19.3%)
**DESIRABLE BMI**			
BMI below 20	1 (1.0%)	6 (4.7%)	7 (3.1%)
BMI 20 – 25	10 (10.0%)	57 (44.5%)	67 (29.4%)
BMI 25 – 30	24 (24.0%)	19 (14.8%)	43 (18.9%)
BMI above 30	2 (2.0%)	3 (2.3%)	5 (2.2%)
Don't know/Not sure/Refused to answer	63 (63.0%)	43 (33.6%)	106 (46.5%)

Desirable BMI (desirable weight/height^2 ^* 703)
